# Adaptive Changes After 2 Weeks of 10-s Sprint Interval Training With Various Recovery Times

**DOI:** 10.3389/fphys.2018.00392

**Published:** 2018-04-17

**Authors:** Robert A. Olek, Sylwester Kujach, Ewa Ziemann, Wieslaw Ziolkowski, Piotr Waz, Radoslaw Laskowski

**Affiliations:** ^1^Department of Bioenergetics and Nutrition, Faculty of Rehabilitation and Kinesiology, Gdansk University of Physical Education and Sport, Gdańsk, Poland; ^2^Department of Physiology, Faculty of Physical Education, Gdansk University of Physical Education and Sport, Gdańsk, Poland; ^3^Department of Physiology and Pharmacology, Faculty of Rehabilitation and Kinesiology, Gdansk University of Physical Education and Sport, Gdańsk, Poland; ^4^Department of Nuclear Medicine, Medical University of Gdańsk, Gdańsk, Poland

**Keywords:** Wingate anaerobic test, all-out exercise, skeletal muscle, enzyme activity, recovery

## Abstract

**Purpose:** The aim of this study was to compare the effect of applying two different rest recovery times in a 10-s sprint interval training session on aerobic and anaerobic capacities as well as skeletal muscle enzyme activities.

**Methods:** Fourteen physically active but not highly trained male subjects (mean maximal oxygen uptake 50.5 ± 1.0 mlO_2_·kg^−1^·min^−1^) participated in the study. The training protocol involved a series of 10-s sprints separated by either 1-min (SIT10:1) or 4-min (SIT10:4) of recovery. The number of sprints progressed from four to six over six sessions separated by 1–2 days rest. Pre and post intervention anthropometric measurements, assessment of aerobic, anaerobic capacity and muscle biopsy were performed. In the muscle samples maximal activities of citrate synthase (CS), 3-hydroxyacylCoA dehydrogenase (HADH), carnitine palmitoyl-transferase (CPT), malate dehydrogenase (MDH), and its mitochondrial form (mMDH), as well as lactate dehydrogenase (LDH) were determined. Analysis of variance was performed to determine changes between conditions.

**Results:** Maximal oxygen uptake improved significantly in both training groups, by 13.6% in SIT10:1 and 11.9% in SIT10:4, with no difference between groups. Wingate anaerobic test results indicated main effect of time for total work, peak power output and mean power output, which increased significantly and similarly in both groups. Significant differences between training groups were observed for end power output, which increased by 10.8% in SIT10:1, but remained unchanged in SIT10:4. Both training protocols induced similar increase in CS activity (main effect of time *p* < 0.05), but no other enzymes.

**Conclusion:** Sprint interval training protocols induce metabolic adaptation over a short period of time, and the reduced recovery between bouts may attenuate fatigue during maximal exercise.

## Introduction

The study by Parolin et al. (1999) described metabolic modifications in skeletal muscle during three 30-s bouts of maximal isokinetic cycling separated by 4-min recovery periods. This exercise protocol has become a basis for sprint interval training (SIT). The studies investigating the effect of SIT have been conducted using three to seven 30-s supramaximal exercise sprint bouts with 4-min rest periods between (Vollaard et al., 2017). Since the aerobic contribution in ATP resynthesis increases in consecutive exercise bouts (Bogdanis et al., 1996; Parolin et al., 1999), SIT is associated with the elevation of muscular oxidative potential as well as central adaptations such as increased cardiac output and stroke volume (Sloth et al., 2013). Consequently, SIT is postulated as a time-efficient exercise strategy for improving maximal oxygen uptake (VO_2_max) (Sloth et al., 2013; Vollaard et al., 2017) with biochemical and morphological adaptations arising in as little as six sessions performed over 2 weeks (Burgomaster et al., 2005, 2006, 2008; Hazell et al., 2010; Lloyd Jones et al., 2017).

It has been suggested that some of the adaptations to SIT are associated with the power generated during the first few seconds of each sprint. Therefore, shorter exercise bouts have been investigated to determine whether resulting adaptations are similar to those observed following 30-s SIT (Hazell et al., 2010; Lloyd Jones et al., 2017). Two various SIT protocols consisting of repeated 30-s or 10-s “all-out” cycle sprints with 4-min rest intervals, performed over 2 weeks indicate comparable enhancement in aerobic and anaerobic exercise capacities (Hazell et al., 2010). Moreover, similar improvements in performance are observed after applying SIT protocols matched for total sprint time −4 × 30-s or 20 × 6-s (Lloyd Jones et al., 2017). Another key factor affecting the metabolic modulations may be the work to rest ratio. Reduction in recovery time seems to be less effective in evoking adaptive changes (Hazell et al., 2010). The increase in aerobic and anaerobic capacities following 10-s SIT with 4-min rest are double changes in 10-s SIT with 2-min rest (Hazell et al., 2010).

Therefore, the aim of the present study was to compare the effect of applying two different rest intervals in 10-s SIT. We hypothesized that reducing the recovery time to 1-min in the SIT protocol consisting of repeated 10-s “all-out” cycle sprints, performed over 2 weeks would be less effective in evoking aerobic adaptations than 10-s SIT with 4-min rest, however it would still improve anaerobic performance measures.

## Materials and methods

**Figure 1:**
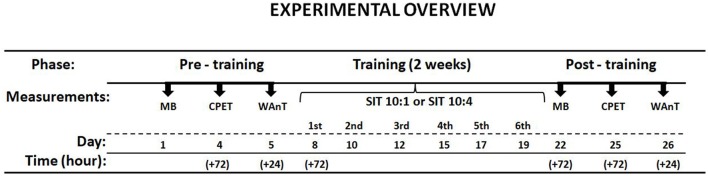
Scheme of the study protocol. Muscle biopsy (MB), cardiopulmonary exercise test (CPET), Wingate anaerobic test (WAnT).

Fourteen physically active but not highly trained male subjects at mean VO_2_max 50.5 ± 1.0 mlO_2_·kg^−1^·min^−1^ volunteered to participate in the study. The subjects were asked to refrain from any additional exercise practice during the study. The study was approved and then completed in accordance with the recommendations of Local Bioethics Committee (http://www.komisjabioetyczna.pl). Written informed consent was obtained from all subjects. The study was conducted in accordance with the Declaration of Helsinki. The overall study protocol is shown in Figure 1. The subjects were assigned to either 1-min interval recovery (SIT10:1) or 4-min interval recovery (SIT10:4) based on the pre-training results.

### Anthropometric measurements

Body mass and composition were estimated using InBody720 (InBody Co., Ltd., Seoul, Korea). Participants were asked to arrive at the laboratory fasted, with voided bladders and bowels. The bioelectrical impedance analyses were performed in the position recommended by the manufacturer guidelines and the subjects clad only in briefs (Ziemann et al., 2013). The impedance measured five segments of the body (arms, trunk, legs) at frequencies of 1, 5, 50, 250, 500, and 1,000 kHz through the eight electrodes. Based on these impedance values fat free mass (FFM) and skeletal muscle mass (SMM) were calculated.

### Cardiopulmonary exercise test

To determine VO_2_max participants performed a graded cycle ergometry test on an electromagnetically-braked, cycle ergometer (ER 900 Jaeger, Viasys Healthcare GmbH, Germany). After a 5-min warm-up at the intensity of 1.5 W·kg^−1^ with a pedaling cadence of 60 rpm, work rate was increased by 25 W·min^−1^ until volitional exhaustion. Breath by breath pulmonary gas exchange was measured by Oxycon-Pro analyzer (Viasys Healthcare GmbH, Germany; Olek et al., 2012). Heart rates were monitored continuously by telemetry (S-625, Polar Electro-Oy, Finland). Maximal heart rate (HRmax), maximal ventilation (VEmax), maximal aerobic power (MAP) were calculated at the VO_2_max level (Ziemann et al., 2011). The anaerobic threshold (AT) has been determined by three independent members of the research group using nonlinear increase in ventilation.

### Anaerobic power measurement

After the standard warm-up, all subjects performed a 30-s “all-out” supramaximal test on a mechanically braked cycle ergometer (884E Sprint Bike, Monark, Sweden). The test was initiated from a dead stop with the resistance equal to 75 g·kg body mass^−1^ (corresponding to 7.5% of an individual's body mass) preset on the ergometer's friction belt (Laskowski et al., 2011). The obtained results were analyzed for total work (TW), peak power output (PPO), mean power output (MPO), end power output (EPO), time to PPO (Bar-Or, 1987). Fatigue index (FI) was determined by taking the percentage difference between PPO and EPO (Richardson et al., 2016).

### Muscle sampling and analyses

Muscle biopsy was made under local anesthesia (2% lidocaine), in the supine position. The sample was obtained by the sterile single-use micro biopsy needle (M.D.L. srl, Delebio, Italy). The section of the *Vastus Lateralis* muscle (~10 mg) was immediately frozen in liquid nitrogen and stored in −80°C until analysis. All biopsies were collected by the same person to ensure a standard localization and muscle depth.

Muscles were homogenized in an ice-cold buffer contained 50 mM potassium phosphate, 1 mM ethylenediaminetetraacetic acid (EDTA), 1 mM threo-1,4-dimercapto-2,3-butanediol at pH 7.4. The homogenates were then centrifuged at 600 g at 4 °C for 10 min. The obtained supernatant was used to determine enzyme activities as previously (Antosiewicz et al., 1995; Kaczor et al., 2005; Olek et al., 2013) with a Super Aquarius CE9200 spectrophotometer (Cecil Instruments Ltd., Cambridge, UK) at 30°C. Citrate synthase (CS) activity was measured by the rate of SH production as CoASH using the thiol reagent 5,5′-dithiobis (2-nitrobenzoic acid) (DTNB). The reagent cocktail contained 50 mM potassium phosphate, 0.1 mM DTNB, and 0.1 mM acetylCoA. The reaction was started by 0.1 mM oxaloacetic acid (OAA). Carnitine palmitoyl-transferase (CPT) activity was measured in the reaction mixture composed by 60 mM of Tris HCl at pH 8.0, 1.5 mM of EDTA with 0.05% Triton X-100 and 0.25 mM DTNB and 1.67 mM of carnitine. The reaction was started by the addition of 0.025 mM palmitoyl-CoA. The kinetics of change in absorbance were followed at 412 nm, and the molar absorption coefficient 14,150 M^−1^cm^−1^ was used for calculation of CS and CPT activities. 3-hydroxyacylCoA dehydrogenase (HADH) activity was determined in a buffer containing 100 mM potassium phosphate and 0.05% Triton at pH 7.4. After addition of supernatant and 0.1 mM NADH the cuvette was preincubated for 3 min. The reaction was started by 0.1 mM acetoacetyl-CoA. Malate dehydrogenase (MDH) activity was determined in the 50 mM Tris-HCl buffer pH 7.6 containing 5 mM EDTA, 0.1 mM NADH, and 0.2 mM OAA. For the mitochondrial MDH (mMDH) assay, the cytoplasmic form of the enzyme (cMDH) was inactivated by 3 min preincubation of homogenate with equal volume of ethanol 99.5% (v/v) at room temperature. Then mMDH activity was followed by the same procedure as for total MDH measurements. Lactate dehydrogenase (LDH) activity was measured in the assay medium contained 50 mM potassium phosphate, 1 mM EDTA, 0.1 mM NADH, 2.1 mM pyruvate, at pH 7.2. The substrates NADH and pyruvate were added immediately before the measurement was started. The change in absorbance was followed in time at 340 nm. HADH, MDH, and LDH activities were calculated using molar absorption coefficient of NADH 6,220 M^−1^ cm^−1^. Protein content was determined by using Bradford protein assay. All reagents were obtained from Sigma-Aldrich.

### Training intervention

Subjects participated in training sessions on Monday, Wednesday, and Friday for 2 weeks. The same research assistant supervised all sessions, controlled the flywheel resistance, as well as the time of exercise and recovery periods. Each training session began with a 5-min warm-up at the intensity approximating 30% MAP. All training was completed using a load of 75 g·kg body mass^−1^.

1. SIT10:1; performed repeated, 10-s “all-out” efforts separated by 1-min recovery.
2. SIT10:4; performed repeated, 10-s “all-out” efforts separated by 4-min recovery.

The number of repeats increased from four repetitions during the first two training sessions, by five repetitions during the middle two training sessions, to six repetitions for the final two training sessions as has been done previously (Hazell et al., 2010). Both training groups performed a similar training protocol consisting of: 30-min low intensity warm-up and 5-min high intensity exercise throughout the training intervention (Stöggl and Sperlich, 2015). The difference between the groups was the time of recovery between the 10-s exercises. Therefore, overall time of training sessions was equal 59-min for SIT10:1 and 131-min for SIT10:4 (Table 1).

**Table 1:** Training distribution over the 2-weeks intervention.

	**SIT10:1**	**SIT10:4**
Total time (min)	59	131
Low intensity (% of total time)	50.8	22.9
High intensity (% of total time)	8.5	3.8
Recovery (% of total time)	40.7	73.3

*The intensity of exercise and its distribution over time have been classified as previously described (Stöggl and Sperlich, 2015)*.

### Statistical analyses

Two-way analysis of variance ANOVA was performed to examine the main effects of group and/or time. In case the ANOVA yielded a significant effect, a Tukey's HSD test was used for *post hoc* comparisons. A probability level *p* < 0.05 was considered statistically significant. Due to the small number of subjects, the effect size (η^2^) has been calculated. The values of η^2^ has been interpreted as follows: 0.1 a small effect, 0.3 a medium effect and 0.5 a large effect, as previously (Olek et al., 2014). All data are expressed as mean ± SEM (standard error of mean).

## Results

The mean body mass of SIT10:4 subjects was higher than SIT10:1 subjects (Table 2), therefore physiological parameters have been presented as relative values. There were no significant differences between groups in aerobic and anaerobic capacities as well as skeletal muscle enzymatic activities at baseline.

**Table 2:** Anthropometric characteristics of participants.

	**SIT10:1**	**SIT10:4**
Age (years)	20.1 ± 0.3	20.7 ± 0.2
Height (cm)	180 ± 1	185 ± 2
Body mass (kg)	75.9 ± 1.7	83.1 ± 2.7^#^
FFM (kg)	66.4 ± 1.5	73.6 ± 2.6^#^
SMM (kg)	37.8 ± 0.9	42.3 ± 1.5^#^
BMI	23.5 ± 0.5	24.3 ± 0.5

*Values are means ± SEM. FFM, fat free mass; SMM, skeletal muscle mass; BMI, body mass index*.

# *p < 0.05 as compared to SIT10:1*.

The training elevated VO_2_max in both groups (Table 3), by 13.6% in SIT10:1 and 11.9% in SIT10:4 (*p* < 0.001, η^2^ = 0.63), with no difference between the groups. Moreover, there was a significant VEmax increase (*p* < 0.05, η^2^ = 0.29) after the training. No significant differences in either HRmax or MAP were observed.

**Table 3:** Aerobic capacities before (pre) and after (post) 2 weeks of SIT.

	**SIT10:1**		**SIT10:4**	
	**Pre**	**Post**	**Pre**	**Post**
**MAX**				
VO_2_ (mlO_2_·min^−1^·kg^−1^)^a^	51.4 ± 1.4	58.4 ± 2.2	49.6 ± 1.5	55.5 ± 2.1
VE (L·min^−1^)^c^	143 ± 6	159 ± 8	152 ± 7	155 ± 6
MAP (W·kg^−1^)	3.9 ± 0.2	4.0 ± 0.2	3.8 ± 0.1	3.9 ± 0.1
HR (bpm)	194 ± 4	191 ± 4	192 ± 1	192 ± 2
**AT**				
VO_2_ (mlO_2_·min^−1^·kg^−1^)^a^	34.0 ± 1.4	39.2 ± 1.2	34.2 ± 1.5	38.2 ± 1.6
VE (L·min^−1^)^a^	67 ± 3	80 ± 3	71 ± 4	77 ± 3
Power (W·kg^−1^)^b^	2.5 ± 0.1	2.7 ± 0.1	2.3 ± 0.1	2.7 ± 0.2
Power (% MAP)^a^	63 ± 3	67 ± 3	62 ± 2	69 ± 2
HR (bpm)	164 ± 4	163 ± 5	154 ± 3	161 ± 4

*Values are means ± SEM. VO_2_, oxygen uptake; VE, minute ventilation; MAP, maximal aerobic power; HR, heart rate*.

a p < 0.001 main training effect;

b p < 0.005 main training effect;

c *p < 0.05 main training effect*.

Significant changes in oxygen uptake (*p* < 0.001, η^2^ = 0.78), VE (*p* < 0.001, η^2^ = 0.74) and workloads (%MAP) (*p* < 0.001, η^2^ = 0.72) at the exercise intensity corresponding to AT intensity following 2 weeks of SIT were observed (Table 3). No significant main effects for group and group × time interaction in these parameters were noted.

**Figure 2:**
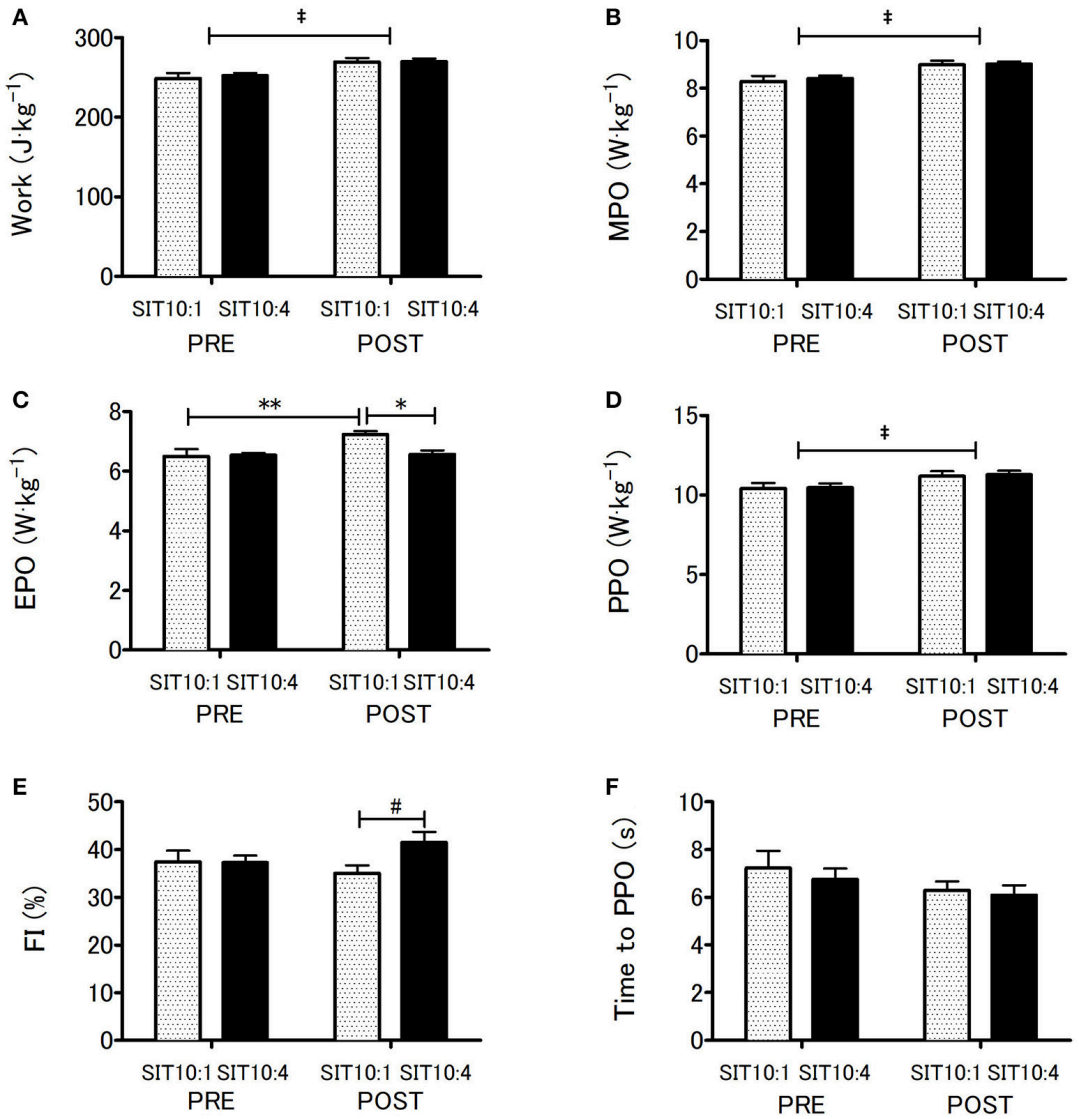
Anaerobic capacity before and after 2 weeks of SIT. Total work **(A)**, mean power output **(B)**, end power output **(C)**, peak power output **(D)**, fatigue index **(E)** and time to peak power output **(F)**. ^‡^*p* < 0.001 main training effect; ^*^p < 0.01 as compared to post-training between the groups; ^**^p < 0.005 as compared to pre-training within the group; ^#^*p* = 0.07 main effect of group.

Wingate anaerobic test results indicated main effect of time for TW (*p* < 0.001, η^2^ = 0.79; Figure 2A), MPO (*p* < 0.001, η^2^ = 0.79; Figure 2B) and PPO (*p* < 0.001, η^2^ = 0.67; Figure 2D) which increased significantly in both groups. However, these changes did not differ between the groups. Significant differences between training groups (*p* < 0.05, η^2^ = 0.32), time (*p* < 0.01, η^2^ = 0.48) and a group x time interaction (*p* < 0.02, η^2^ = 0.39) were recorded for EPO (Figure 2C). EPO in SIT10:1 increased significantly by 10.8% (*p* < 0.005), whereas in SIT10:4 group remained unchanged (*p* = 0.9). Changes in EPO affected FI, which approached statistical significance after the training (*p* = 0.07, η^2^ = 0.25; Figure 2E). No differences in time to PPO was noted (Figure 2F).

There was a main effect of time for CS activity, which elevated significantly in both training groups (*p* < 0.05, η^2^ = 0.33), but no for HADH (*p* = 0.18, η^2^ = 0.14), mMDH (*p* = 0.17, η^2^ = 0.15) and CPT (*p* = 0.9, η^2^ = 0.0). There were no differences in LDH activity and LDH/CS ratio (Table 4).

**Table 4:** Maximal activities of skeletal muscle enzymes before (pre) and after (post) 2 weeks of SIT.

	**SIT10:1**		**SIT10:4**	
	**Pre**	**Post**	**Pre**	**Post**
CPT (mU·mg protein^−1^)	1.6 ± 0.1	1.6 ± 0.1	1.7 ± 0.1	1.7 ± 0.1
CS (mU·mg protein^−1^)^a^	161 ± 19	193 ± 13	177 ± 13	202 ± 13
HADH (mU·mg protein^−1^)	116 ± 11	132 ± 5	114 ± 11	123 ± 12
MDH (U·mg protein^−1^)	5.2 ± 0.7	5.4 ± 0.5	4.9 ± 0.5	5.9 ± 0.6
cMDH (U·mg protein^−1^)	3.9 ± 0.5	4.0 ± 0.3	3.7 ± 0.4	4.4 ± 0.4
mMDH (U·mg protein^−1^)	1.3 ± 0.2	1.4 ± 0.1	1.2 ± 0.1	1.5 ± 0.2
LDH (U·mg protein^−1^)	3.2 ± 0.4	3.8 ± 0.2	3.6 ± 0.5	3.8 ± 0.3
LDH/CS	20.3 ± 0.8	20.2 ± 2.1	20.8 ± 2.5	19.6 ± 2.6

*Values are means ± SEM. CPT, carnitine palmitoyl-transferase; CS, citrate synthase; HADH, 3-hydroxyacylCoA dehydrogenase; MDH, malate dehydrogenase; cMDH, cytoplasmic malate dehydrogenase; mMDH, mitochondrial malate dehydrogenase; LDH, lactate dehydrogenase*.

a *p < 0.05 main training effect*.

## Discussion

The main finding of this study is that both 1-min and 4-min recovery intervals in repeated 10-s sprint intervention for 2 weeks, resulted in a similar improvements in aerobic (VO_2_max, AT) and anaerobic (TW, PPO, MPO) capacities, and skeletal muscle enzyme activities. Moreover, the shorter recovery time induced higher EPO and lower FI during Wingate anaerobic test.

The increase in VO_2_max is similar to data obtained by McGarr et al. (2014) and Richardson and Gibson (2015), who used 30-s sprints with 4-min recoveries in training protocols performed over 2 weeks, reporting 14.2 and 11.2% improvement, respectively. Hazell et al. (2010), by applying six SIT sessions consisting of 10-s sprints with 4-min recovery intervals, indicated an 8.5% enhancement in aerobic capacity, similar to 30-s “all-out” cycles interspersed with 4-min recoveries (8.3%) reported in the same study. However, the workload used during SIT was slightly higher (10% of individual's body mass) (Hazell et al., 2010) than in the current protocol (7.5% of individual's body mass). On the contrary, two other studies applying 10-s maximal cycling SIT reported no change in VO_2_max: 2.4% (Hellsten-Westing et al., 1993) and −1.3% (Skleryk et al., 2013). These discrepancies may be caused by the number of repetitions applied during SIT (Vollaard et al., 2017). SIT performed by Hellsten-Westing et al. (1993) consisted of 15 10-s maximal sprints, three times per week for a total of 6 weeks. Skleryk et al. (2013) implemented six SIT sessions of 10-s “all-out” cycling, repeated 8–12 times. In the recent meta-analysis, Vollaard et al. (2017) indicated that training protocols consisting of fewer repetitions during session induced greater VO_2_max increase. Authors negated the role of total energy use, energy turnover or oxygen transfer in improvement of VO_2_max with SIT, because for each of these factors the stimulus should be greater with more sprint repetitions (Vollaard et al., 2017).

Our results revealed that 1-min or 4-min rest periods did not impact the increase in relative values of VO_2_max. Both training protocols were characterized by supramaximal intensity. Despite being short-lasting, both had a positive effect on maximal ventilation values, allowing subjects to perform the high volume workload. Moreover, maximal oxygen uptake also strongly correlates with skeletal muscle mitochondrial capacities (Rasmussen et al., 2001). SIT-induced VO_2_max improvement is associated with changes in mitochondrial bioenergetics (Larsen et al., 2013). Six SIT sessions of 30-s sprints, performed over 2 weeks caused maximal CS activity elevation (Burgomaster et al., 2005, 2006). On the other hand, HADH, the rate-limiting fat oxidation mitochondrial enzyme, does not change after 2 weeks (Burgomaster et al., 2006), however, it significantly increases after 6 weeks of 30-s SIT (Burgomaster et al., 2008). Higher HADH has also been observed in legs, but not in arms, following 72 “all-out sprints” (36 with arm cycling and 36 with leg cycling separated by 1-h of recovery) during the seven training sessions (Zinner et al., 2016). We have shown that a SIT protocol consisting the same number of repetitions as previously reported (Burgomaster et al., 2005, 2006), although with lower total work done, induced a significant rise in CS activity (but not in other mitochondrial enzymes). Since training induced changes in muscle CS activity are matched by changes in whole body oxidative capacity (Vigelsø et al., 2014; Meinild Lundby et al., 2018), our results confirm the effectiveness of both SIT protocols in training-induced adaptations.

Elevated oxidative ATP synthesis, attenuates the contribution of anaerobic ATP production (Larsen et al., 2014) and delays the blood lactate accumulation (Jakeman et al., 2012) following SIT. We have not determined lactate production, but we have observed changes in maximal oxygen uptake and CS activity, which were associated with higher workload at the intensity corresponding to AT. In addition, aerobic/anaerobic energy supply depends on the rate of pyruvate production and its mitochondrial oxidative decarboxylation catalyzed by pyruvate dehydrogenase competing to cytosolic reduction to lactate via LDH (Spriet et al., 2000). Moreover, malate-aspartate shuttle appears to be quantitatively important in lactate production during exercise (Schantz et al., 1986). Although the training protocol applied in our study did not modify LDH activity, and the increase in MDH activity was not statistically significant, we have observed higher workout at the intensity corresponding to AT, suggesting reduced lactate production following SIT.

SIT performed daily for 2 weeks increases skeletal muscle phosphocreatine (PCr) content and creatine kinase (CK) activity, but not PPO nor MPO during the Wingate test (Parra et al., 2000; Rodas et al., 2000). On the contrary, the same 14 sessions applied for 6 weeks (resting for 2 days between each session) causes improvement in PPO and MPO with no change in PCr and CK (Parra et al., 2000). PPO increases with no modification in PCr also after completing only 6 SIT sessions over 2 weeks (Burgomaster et al., 2005). Similarly, higher PPO has been reported in a study utilizing 6 sessions of 10-s supramaximal bouts, with 2-min or 4-min recoveries between sprints (Hazell et al., 2010). Consistently, we observed a significant PPO increase in both SITs. Despite no differences between the two protocols in other measured parameters, we observed an improvement in EPO during Wingate test in the group with a shorter recovery time.

Aerobic energy provision contributes almost half of the ATP turnover during the repeated 30-s sprint (after 4-min recovery; Bogdanis et al., 1996). Hence, the adaptive changes in oxidative metabolism seem to be reasonable following such a training protocol. During the 10-s sprint, the PCr availability is important for high power output (Bogdanis et al., 1998). Since PCr resynthesis 2-min after cessation of exercise reaches about ~90% of the resting value (Hultman et al., 1967; Bogdanis et al., 1998), subjects are able to reproduce PPO after 2-min recovery (Bogdanis et al., 1998). It seems that the reported adaptations following such training protocols may be caused by increased flux in the creatine-PCr energy shuttle (Bessman and Carpenter, 1985). Bessman and Savabi (1988) suggested that creatine plays important role in high energy phosphate transport, necessary for protein synthesis, ion transport and muscle contraction. The PCr resynthesis 1-min and 4-min after the exercise is ~80 and 90% (Hultman et al., 1967), consequently the small difference seems to be insufficient to induce various adaptive response for two SIT protocols. On the other hand, accumulation of incomplete recoveries in 1-min recovery SIT may contribute to some adaptations in ionic regulation resulting in EPO improvement. However, an increasing number of sprints may accumulate fatigue (Vollaard et al., 2017) and thus the effectiveness of training protocol in aerobic adaptations may be reduced (Hellsten-Westing et al., 1993; Skleryk et al., 2013).

## Conclusion

This study found that 2 weeks of SIT comprising either of 1- or 4-min recovery time between exercises, which were matched for total sprint time, elicited similar performance changes in fit, healthy men. Moreover, reduced time of recovery between bouts may be more effective in attenuating fatigue during maximal exercise. Overall, obtained data complement presently available knowledge about interactions between intensity as well as duration of interval protocols and recovery. This is particularly significant in the context of the latest recommendations of American College of Sports Medicine; which cites high/sprint intensity interval training as the most effective form of exercise (Thompson, 2017).

## Ethics statement

This study was carried out in accordance with the recommendations of Local Bioethics Committee with written informed consent from all subjects. All subjects gave written informed consent in accordance with the Declaration of Helsinki. The protocol (Figure 1) was approved by the Local Bioethics Committee (http://www.komisjabioetyczna.pl/).

## Author contributions

RO, SK, and RL: Conceived and design the experiment; RO, SK, EZ, WZ, and RL: Performed the data collection; RO and PW: Performed the statistical analysis and interpretation of data; RO, SK, EZ, WZ, PW, and RL: Participated in drafting the article or revising it critically for important intellectual content; RO, SK, EZ,WZ, PW, and RL: Approved the final manuscript.

### Conflict of interest statement

The authors declare that the research was conducted in the absence of any commercial or financial relationships that could be construed as a potential conflict of interest.

## Abbreviations

SIT: sprint interval training
VO_2_max: maximal oxygen uptake
VE: minute ventilation
HR: heart rate
MAP: maximal aerobic power
AT: anaerobic threshold
TW: total work
PPO: peak power output
MPO: mean power output
EPO: end power output
FI: fatigue index
FFM: fat free mass
SMM: skeletal muscle mass
BMI: body mass index
CS: citrate synthase
HADH: 3-hydroxyacylCoA dehydrogenase
CPT: carnitine palmitoyl-transferase
MDH: malate dehydrogenase
mMDH: mitochondrial malate dehydrogenase
cMDH: cytoplasmic malate dehydrogenase
LDH: lactate dehydrogenase
EDTA: ethylenediaminetetraacetic acid
DTNB: 5,5′-dithiobis (2-nitrobenzoic acid)
OAA: oxaloacetic acid
NADH: nicotinamide adenine dinucleotide reduced form.
